# Challenges in achieving a target international normalized ratio for deep vein thrombosis among HIV-infected patients with tuberculosis: a case series

**DOI:** 10.1186/s12878-016-0056-6

**Published:** 2016-06-04

**Authors:** C Sekaggya, D Nalwanga, A Von Braun, R Nakijoba, A Kambugu, J Fehr, M Lamorde, B Castelnuovo

**Affiliations:** Infectious Diseases Institute, College of Health Sciences, Makerere University, P.O. Box 22418, Kampala, Uganda; Division of Infectious Diseases and Infection Control, University Hospital Zurich, University of Zurich, Zurich, Switzerland

**Keywords:** Tuberculosis, HIV, Thrombosis, Monitoring

## Abstract

**Background:**

Tuberculosis (TB) and HIV are among the risk factors for deep vein thrombosis (DVT). There are several challenges in the management of DVT patients with TB-HIV co-infection including drug-drug interactions and non-adherence due to pill burden.

**Methods:**

HIV infected patients starting treatment for TB were identified and followed up two weekly. Cases of DVT were diagnosed with Doppler ultrasound and patients were initiated on oral anticoagulation with warfarin and followed up with repeated INR measurements and warfarin dose adjustment.

**Results:**

We describe 7 cases of TB and HIV-infected patients in Uganda diagnosed with DVT and started on anticoagulation therapy. Their median age was 30 (IQR: 27–39) years and 86 % were male. All patients had co-medication with cotrimoxazole, tenofovir, lamivudine and efavirenz and some were on fluconazole. The therapeutic range of the International Normalization Ratio (INR) was difficult to attain and unpredictable with some patients being under-anticoagulated and others over-anticoagulated. The mean Time in Therapeutic Range (TTR) for patients who had all scheduled INR measurements in the first 12 weeks was 33.3 %. Only one patient among those with all the scheduled INR measurements had achieved a therapeutic INR by 2 weeks. Four out of seven (57 %) of the patients had at least one INR above the therapeutic range which required treatment interruption. None of the patients had major bleeding.

**Conclusion:**

We recommend more frequent monitoring and timely dose adjustment of the INR, as well as studies on alternative strategies for the treatment of DVT in TB-HIV co-infected patients.

## Background

The risk of deep vein thrombosis (DVT) is one and a half times higher among patients with tuberculosis (TB) compared to those without TB [1] due to a hypercoagulable state which occurs among patients with TB resulting from endothelial dysfunction due to the mycobacteria, increased fibrinogen, fibrin, tissue plasminogen activator, decreased anti-thrombin III [2, 3] and use of rifampicin especially in the first two weeks following TB treatment initiation [4]. Furthermore HIV infection, which is the highest risk factor for TB, is also considered a pro-thrombotic condition occurring most commonly in those with a low CD4 cell count [5, 6]. The mechanism leading to DVT in HIV is thought to be multifactorial including Protein C, S and antithrombin deficiency and increased antiphospholipid and antilupus antibodies [7, 8].

Several challenges due to drug-drug interactions occur during the management of DVT in patients co-infected with HIV and TB. Warfarin is metabolized by CYP450 pathway. Therefore drugs which induce this pathway (rifampicin and nevirapine) or inhibit them (isoniazid and efavirenz) could lead to under-anticoagulation or over-anticoagulation respectively. These interactions influence the ability to attain the therapeutic target required for adequate anticoagulation [9]. Table 1 demonstrates drug interactions that may occur in patients with HIV and TB.

**Table 1:** Drug interactions in patients with HIV and TB

DRUG	EFFECT ON CYTOCHROME P450	
	Induce Cytochrome P450 (Decrease effect of warfarin)	Inhibit Cytochrome P450 (Increase effect of warfarin)
Rifampicin	X	
Ritonavir (protease inhibitor)	X	
Nevirapine	X	
Isoniazid		X
Efavirenz^a^	X	X
Fluconazole		X
Cotrimoxazole		X

^a^Efavirenz may induce or inhibit cytochrome P450 through its action on CYP3A4 and CYP2C9 respectively

Several case series have discussed the occurrence of thrombosis in patients with TB [10, 11] and in those with HIV [12, 13], however, in this case series we demonstrate the challenges encountered when trying to achieve an International Normalized Ratio (INR) within the therapeutic window with warfarin in this population of TB-HIV co-infected patients.

## Methods

The cases described are from patients enrolled in the “Study of Outcomes in TB-HIV co-infected patients” (SOUTH), a study conducted at the integrated HIV and TB clinic at the Infectious Diseases Institute in Kampala [14]. The SOUTH study aims to investigate the relationship between anti-TB drug concentrations and treatment outcomes in HIV infected patients treated for pulmonary TB (PTB). Patients are reviewed by a clinician every two weeks during the first two months of their treatment and monthly subsequently. Patients with clinical symptoms of TB are investigated using chest x-ray, sputum smear microscopy, sputum culture for *Mycobacterium tuberculosis* and Xpert MTB/RIF. Patients were followed up starting from the day TB treatment was initiated. TB treatment included a fixed dose regimen consisting of two months of rifampicin, isoniazid, pyrazinamide and ethambutol followed by four months of rifampicin and isoniazid. CD4 counts were measured within two weeks prior to or after TB diagnosis. Patients were initiated on antiretroviral therapy (ART) after the second week of anti-TB treatment according to WHO guidelines [15, 16] and included tenofovir, lamivudine and efavirenz. Patients remained on this ART regimen throughout the follow-up period. All patients were on cotrimoxazole before the start of the study and also remained on it throughout follow-up. Cases of DVT were identified by clinical history and physical examination between May 2013 and June 2015; all patients who reported or were observed to have limb swelling were referred for Doppler ultrasound scan to confirm the clinical diagnosis. Patients diagnosed with DVT were initiated on warfarin tablets (Bristol®) at an initial dose of 2.5 - 5 mg once daily, as well as low molecular heparin (LMWH), Enoxaparin (Clexane®) 1 mg/kg for five days and subsequently continued on warfarin alone. The INR was monitored weekly and dose adjustment was made at the discretion of the clinician depending on the INR results. Adherence to warfarin was assessed through self-report and the number of days that warfarin doses were missed were recorded in the patient’s file. Patients who missed a visit were called on the same day and rescheduled for the closest opportunity within the same week. Time in therapeutic range (TTR) was calculated as the number of therapeutic INR values during the first 12 weeks of anticoagulation as a percentage of all the INR values measured during this same period.

Informed consent was obtained from all patients prior to involvement in the study.

The study was reviewed and approved by the Joint Clinical Research Centre Research and Ethics Committee and the Uganda National Council for Science and Technology (HS 1303).

## Results

During this review period, 7/268 (2.6 %) patients with confirmed PTB presented with pain and swelling of the lower limb and were diagnosed with DVT through Doppler ultrasound scan. All patients were HIV positive. Individual patients’ characteristics are displayed in Table 2. Six (86 %) were male with a median age of 30 (interquartile range (IQR): 27–39) years and a median CD4 count at the time of TB diagnosis of 72cells/μl (19–78). All patients were not on ART at the time of anti-TB treatment initiation and started on tenofovir, lamivudine and efavirenz after two weeks of TB treatment. The median time from initiation of anti-TB treatment to DVT diagnosis was 2 (IQR: 2–4) weeks. From their clinical history, none of the patients was bedridden at the time of DVT diagnosis.

**Table 2:** Patient baseline characteristics

Patient number	Age (years)	Gender	Baseline CD4 (cells/μL)	Week of TB treatment when DVT diagnosed	Symptoms of bleeding	Co-medication^a^
1	30	M	6	1.5	No	CTX, Fluconazole
2	26	M	73	4	No	CTX
3	40	F	40	2	Epistaxis	CTX
4	39	M	72	10	No	CTX
5	27	M	78	2	Epistaxis	CTX, fluconazole
6	31	M	19	4	No	CTX
7	28	M	180	2	No	CTX, fluconazole

*M* male, *F* female, *CTX* cotrimoxazole

^a^All patients were on tenofovir, lamivudine and efavirenz

**Fig. 1:**
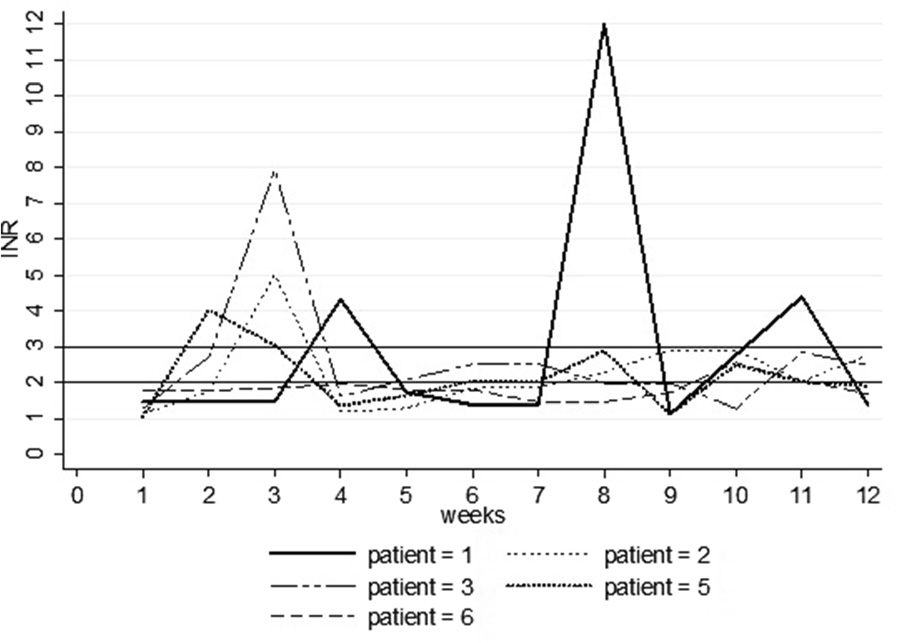
INR trends during the first 12 weeks of anticoagulation

Three patients were on 600-800 mg of fluconazole before the diagnosis of DVT was made (patients 1, 5 and 7) due to cryptococcal antigenemia. Figure 1 below shows the trend of INR values for each patient while Table 3 shows the corresponding warfarin doses.

**Table 3:** Warfarin doses adjustments per week

Patient number	Dose of warfarin (mg)											
	Week 1	Week 2	Week 3	Week 4	Week 5	Week 6	Week 7	Week 8	Week 9	Week 10	Week 11	Week 12
Patient 1	5	7.5	7.5	7.5	0	5	7.5	10	0	5	5	0
Patient 2	5	7.5	10	0	5	7.5	7.5	10	10	10	10	10
Patient 3	5	10	10	0	2.5	5	5	5	7.5	7.5	7.5	7.5
Patient 5	2.5	5	0	0	5	5	5	5	5	5	5	5
Patient 6	2.5	5	5	7.5	7.5	10	10	12.5	12.5	12.5	12.5	12.5

Patient 1 was started on the standard ART mentioned ten days after the diagnosis of DVT was made. He had only one therapeutic INR during the first 12 weeks of anticoagulation with a TTR of 8.3 %. Three of his INR measurements were supratherapeutic with no major bleeding while taking 7.5 mg and 5 mg respectively which were initially leading to sub-therapeutic INR values. His maximum dose during this period was 10 mg. He was on fluconazole throughout the follow-up period.

Patient 2, who had started ART two weeks prior to the diagnosis of DVT had a supratherapeutic INR after 3 weeks of anticoagulation while on 10 mg of warfarin with no major bleeding episode. From 8 weeks onwards, his INR was maintained within the therapeutic range on 10 mg. His TTR was 41.7 %.

Patient 3 had a therapeutic INR at the earliest time point compared to the other patients (1 week) and had the best INR control. She developed DVT two weeks into her TB treatment and ART and warfarin were started at the same time. She had a supratherapeutic INR while on 10 mg of warfarin with epistaxis. She later had a therapeutic INR initially on 5 mg and later 7.5 mg of warfarin. She had the highest TTR of 58.3 %.

Patient 4 had a history of alcohol abuse and had five intermittent INR measurements (1.53 at week one, 2.10 at week two, 1.29 at week six, 1.04 at week ten and 1.29 at week twelve) He missed visits at weeks 3, 4, 5, 7, 8, 9, 11 despite phone call reminders to come to the clinic. He admitted to poor adherence whenever he run out of pills.

Patient 5 started on ART on the day DVT was diagnosed, and was also on fluconazole. He had supratherapeutic INR on a dose of 5 mg of warfarin. This same dose subsequently kept him within the therapeutic range for most of the time after week 6 even while he was still on fluconazole and his TTR was 41.7 %.

Patient 6 was diagnosed with DVT a week after starting ART. He had dose escalation up to 12.5 mg, the highest dose used among these patients, and only attained a therapeutic INR at two time points. He attained the lowest TTR of 16.7 %.

Patient 7 had only 2 INR measurements after 1 and 3 weeks (1.15 and 2.21 respectively) and there after requested to be transferred to another center.

The mean TTR for patients who had all scheduled INR measurements in the first 12 weeks was 33.3 %. Only one patient (patient 3) among those with all the scheduled INR measurements had achieved a therapeutic INR by 2 weeks.

Four out of seven (57 %) of the patients (cases 1, 2, 3 and 5) had at least one INR above the therapeutic range which required treatment interruption. Two patients developed epistaxis (patient 5 and 3); however none of them experienced severe bleeding. Patient 1 had supratherapeutic INR values at weeks 4, 8 and 11 and the INR only reached the therapeutic range once during the first 12 weeks of treatment despite several dose adjustments each week as shown in the graph below.

Of the remaining two patients, one (patient 7) missed several clinic visits and subsequently asked to be transferred to another health care center and patient 4 frequently missed clinic visits and achieved therapeutic ranges only at time point (week 4). All patients reported 100 % adherence except patients 4 and 7 who missed over 60 % of their warfarin doses during the follow-up period.

## Discussion

Achieving a target INR was challenging in our TB-HIV co-infected population, and we supposed that drug-drug interactions and unreported non-adherence could have played a role. Several dose adjustments occurred which resulted in supratherapeutic or subtherapeutic INR values. On some occasions, the same dose of warfarin that kept a patient within the therapeutic range would also lead to a subtherapeutic or supratherapeutic INR later as in the case of patients 1 and 2 which could be due to drug interactions or non-adherence that was not reported.

Warfarin prescriptions increase the pill burden in TB-HIV co-infected patients who are already taking up to 9 tablets per day. Pill burden has been shown to enhance non-adherence [17] and therefore contribute to difficulty in attaining a target INR.

Drug interactions with fluconazole, which increases the effect of warfarin through inhibition of CYP2C9 and CYP3A4, may explain the low daily warfarin requirement for patients 1 and 5 compared to the other patients. Patient 1 also had several supratherapeutic INR values due to the increased effect of warfarin. Drug-drug interactions between warfarin and fluconazole has also been reported by others [18].

All patients were on rifampicn, isoniazid and efavirenz, making it difficult to ascertain which of these drugs contributed to the subtherapeutic or supratherapeutic INR values; however, the challenge of drug-drug interactions is evident. Lee et al. reported that a 233 % increase in warfarin dosage over 4 months was insufficient to attain an INR within the therapeutic range in a patient on rifampicin [19].

Where there are drug interactions, a switch to a more suitable option is necessary, however we recognize that there are not many alternative therapies for treatment of DVT; therefore this may not be practical. Newer drugs like factor Xa inhibitors also pose a challenge due to drug-drug interactions in patients on TB treatment and ART as is the case of warfarin. LMWH which is the recommended anticoagulation therapy in patients with cancer, may be suitable for this patient population, an area that also needs to be explored further considering the similar mechanisms of thrombosis involving an increase in inflammatory markers.

Most INR values measured in these patients were outside the therapeutic range and the mean time in the therapeutic range for all the patients with regular follow-up was only 33.3 %. Although we did not have a direct measure of clinical improvement, TTR has been shown to correlate with DVT treatment outcome and is used in the assessment of the management of DVT [20]. There are however many methods of measurement of TTR which may limit its utility in day to day practice. Time in therapeutic range has been reported to be higher in other studies (56 %–75 %) [21] in comparison to our patients.

Our time in therapeutic range may have implications on the duration of treatment that is adequate to achieve actual resolution of symptoms and resorption of the thrombus. Further studies need to evaluate if prolonging treatment in patients with low TTR values affects DVT treatment outcomes.

## Conclusion

The course of INR in this population was unpredictable in any individual patient. This could be due to a number of reasons including non-adherence and changing dose requirements due to drug interactions.

More frequent monitoring of INR with timely dose adjustments is required in TB-HIV patients. Studies on alternative medication for example long term LMWH in TB-HIV infected patients should be considered.

## Abbreviations

ART, Antiretroviral therapy; CD4, Cluster of Differentiation; CTX, Cotrimoxazole; DVT, Deep vein thrombosis; HIV, Human Immunodeficiency virus; INR, International Normalized Ratio; IQR, Interquartile range; MTB/Rif, Mycobacteria tuberculosis/Rifampicin; TB, Tuberculosis; TTR, Time in therapeutic range.

## References

[1] Dentan C, Epaulard O, Seynaeve D, Genty C, Bosson JL (2014). Active tuberculosis and venous thromboembolism: association according to international classification of diseases, ninth revision hospital discharge diagnosis codes. Clin Infect Dis.

[2] Robson SC, White NW, Aronson I, Woollgar R, Goodman H, Jacobs P (1996). Acute-phase response and the hypercoagulable state in pulmonary tuberculosis. Br J Haematol.

[3] Kager LM, Blok DC, Lede IO, Rahman W, Afroz R, Bresser P (2015). Pulmonary tuberculosis induces a systemic hypercoagulable state. J Infect.

[4] White NW (1989). Venous thrombosis and rifampicin. Lancet.

[5] Sullivan PS, Dworkin MS, Jones JL, Hooper WC (2000). Epidemiology of thrombosis in HIV-infected individuals. The Adult/Adolescent Spectrum of HIV Disease Project. AIDS.

[6] Rasmussen LD, Dybdal M, Gerstoft J, Kronborg G, Larsen CS, Pedersen C (2011). HIV and risk of venous thromboembolism: a Danish nationwide population-based cohort study. HIV Med.

[7] Leder AN, Flansbaum B, Zandman-Goddard G, Asherson R, Shoenfeld Y (2001). Antiphospholipid syndrome induced by HIV. Lupus.

[8] Majluf-Cruz A, Silva-Estrada M, Sanchez-Barboza R, Montiel-Manzano G, Trevino-Perez S, Santoscoy-Gomez M (2004). Venous thrombosis among patients with AIDS. Clin Appl Thromb Hemost.

[9] Liedtke MD, Rathbun RC (2009). Warfarin-antiretroviral interactions. Ann Pharmacother.

[10] Goncalves IM, Alves DC, Carvalho A, Do Ceu Brito M, Calvario F, Duarte R (2009). Tuberculosis and Venous Thromboembolism: a case series. Cases J.

[11] Kumarihamy KW, Ralapanawa DM, Jayalath WA (2015). A rare complication of pulmonary tuberculosis: a case report. BMC Res Notes.

[12] Jacobson MC, Dezube BJ, Aboulafia DM (2004). Thrombotic complications in patients infected with HIV in the era of highly active antiretroviral therapy: a case series. Clin Infect Dis.

[13] Konin C, Anzouan-Kacou JB, Essam N’l A (2011). Arterial thrombosis in patients with human immunodeficiency virus: two-case reports and review of the literature. Case Rep Vasc Med.

[14] Hermans SM, Castelnuovo B, Katabira C, Mbidde P, Lange JM, Hoepelman AI (1999). Integration of HIV and TB services results in improved TB treatment outcomes and earlier prioritized ART initiation in a large urban HIV clinic in Uganda. J Acquir Immune Defic Syndr.

[15] Padayatchi N, Abdool Karim SS, Naidoo K, Grobler A, Friedland G (2014). Improved survival in multidrug-resistant tuberculosis patients receiving integrated tuberculosis and antiretroviral treatment in the SAPiT Trial. Int J Tuberc Lung Dis.

[16] WHO (2012). Policy on Collaborative TB/HIV Activities: Guidelines for National Programmes and Other Stakeholders.

[17] Kneeland PP, Fang MC (2010). Current issues in patient adherence and persistence: focus on anticoagulants for the treatment and prevention of thromboembolism. Patient Preference Adherence.

[18] Gericke KR (1993). Possible interaction between warfarin and fluconazole. Pharmacotherapy.

[19] Lee CR, Thrasher KA (2001). Difficulties in anticoagulation management during coadministration of warfarin and rifampin. Pharmacotherapy.

[20] Phillips KW, Ansell J (2008). Outpatient management of oral vitamin K antagonist therapy: defining and measuring high-quality management. Expert Rev Cardiovasc Ther.

[21] Erkens PM, Ten Cate H, Buller HR, Prins MH (2012). Benchmark for time in therapeutic range in venous thromboembolism: a systematic review and meta-analysis. PLoS One.

